# Optical probing of gastrocnemius in patients with peripheral artery disease characterizes myopathic biochemical alterations and correlates with stage of disease

**DOI:** 10.14814/phy2.13161

**Published:** 2017-03-14

**Authors:** Ryan A. Becker, Kim Cluff, Nithyanandhi Duraisamy, Hootan Mehraein, Hussam Farhoud, Tracie Collins, George P. Casale, Iraklis I. Pipinos, Jeyamkondan Subbiah

**Affiliations:** ^1^ Biomedical Engineering DepartmentWichita State UniversityWichitaKansas; ^2^ Industrial EngineeringWichita State UniversityWichitaKansas; ^3^ Heartland CardiologyWichitaKansas; ^4^ Department of Preventive Medicine & Public HealthSchool of MedicineUniversity of Kansas Medical CenterWichitaKansas; ^5^ Division of General SurgeryDepartment of SurgeryUniversity of Nebraska Medical CenterOmahaNebraska; ^6^ Department of Surgery and VA Research ServiceVA Nebraska‐Western Iowa Health Care SystemOmahaNebraska; ^7^ Biological Systems Engineering and Food Science and TechnologyUniversity of Nebraska‐LincolnLincolnNebraska

**Keywords:** Atherosclerosis, *ATR‐FTIR* Spectroscopy, ischemia, muscle damage, vascular disease

## Abstract

Peripheral artery disease (PAD) is a condition caused by atherosclerotic blockages in the arteries supplying the lower limbs and is characterized by ischemia of the leg, progressive myopathy, and increased risk of limb loss. The affected leg muscles undergo significant changes of their biochemistry and metabolism including variations in the levels of many key proteins, lipids, and nucleotides. The mechanisms behind these changes are poorly understood. The objective of this study was to correlate the severity of the PAD disease stage and associated hemodynamic limitation (determined by the ankle brachial index, ABI) in the legs of the patients with alterations in the biochemistry of chronically ischemic leg muscle as determined by ATR‐Fourier transform infrared micro‐spectroscopy. Muscle (gastrocnemius) biopsies were collected from 13 subjects including four control patients (ABI≥0.9), five claudicating patients (0.4 ≤ ABI<0.9), and four critical limb ischemia (CLI) patients (ABI<0.4). Slide mounted specimens were analyzed by ATR‐Fourier transform infrared micro‐spectroscopy. An analysis of variance and a partial least squares regression model were used to identify significant differences in spectral peaks and correlate them with the ABI. The spectra revealed significant differences (*P* < 0.05) across control, claudicating, and CLI patients in the fingerprint and functional group regions. Infrared microspectroscopic probing of ischemic muscle biopsies demonstrates that PAD produces significant and unique changes to muscle biochemistry in comparison to control specimens. These distinctive biochemical profiles correlate with disease progression and may provide insight and direction for new targets in the diagnosis and therapy of muscle degeneration in PAD.

## Introduction

Peripheral artery disease (PAD) is a consequence of reduced blood flow caused by atherosclerotic plaque buildup, (Schirmang et al. 2009) causing stenosis and obstruction in the arteries supplying the lower limbs (Flu et al. 2010). The most common and early presenting symptom of PAD is claudication, which is usually identified by muscle discomfort, fatigue or pain in the legs after walking a short distance (Schirmang et al. 2009). If the disease continues to progress, blood flow to the extremities continues to decrease, causing critical limb ischemia (CLI), a condition characterized by chronic ischemic pain at rest and tissue loss (nonhealing ulcers or gangrene). At this stage of the disease, the prognosis of the limb is poor, with half of patients presenting with CLI requiring limb amputation within 1 year from their presentation (Feinglass et al. 1999; Norgren et al. 2007; Ziegler‐Graham et al. 2008). Metabolic demands of the ischemic tissue, the location of the affected artery, and the degree of the myopathy in the leg muscles are all key factors that influence the severity of PAD symptoms (Pipinos et al. 2007, 2008; Hills et al. 2009).

Currently the ankle‐brachial index (ABI), a simple noninvasive technique for detecting arterial obstructions, is used as the standard test for PAD diagnosis (McLafferty et al. 1997). Arterial blockages are identified by dividing the systolic blood pressure at each ankle by the systolic blood pressure at the arm, and the ratio of these values is used to determine the degree of restriction of blood flow through the lower extremities (McLafferty et al. 1997). Other noninvasive diagnostic tests include pulse volume recordings, toe brachial index, and transcutaneous oxygen measurement (McLafferty et al. 1997). However, none of these tests measure the effects that reduced blood flow has on the end organ and the damage to the skeletal muscle. At the histological level, progressive skeletal muscle damage causes structural abnormalities in the myofibers, as well as intracellular‐extracellular histological and biochemical changes (Busch et al. 1972; Cullen and Fulthorpe 1975; Cluff et al. 2013). These changes indicate damage at a level which cannot be measured by the ABI and its related tests (Maunder et al. 1977).

Recently, optical probing methods, such as Raman spectroscopy, Fourier Transform Infrared (FTIR) spectroscopy, and hyperspectral imaging have shown promise toward providing important molecular information for the analysis of diseases. These techniques are novel because they utilize label‐free methods to obtain information‐rich biochemical spectral signatures of diseased tissues, providing insight into the disease progression that cannot be identified through traditional histological analysis. Prior work from our group has shown that Raman spectroscopy can identify key changes in spectral peaks which correlate with hemodynamic limitation (ABI) in PAD patients (Cluff et al. 2014). Fourier transform infrared (FTIR) spectroscopy is an analytical technique that measures changes in molecular vibrations within a tissue sample, and is a powerful tool that can be used to characterize the biochemical profile at the cellular level for many pathologies (Wood et al. 1996; Wang et al. 1997; Eckel et al. 2001; Fujioka et al. 2004; Baker et al. 2008). Further, it is considered complimentary to Raman spectroscopy, providing information from infrared (IR) active peaks that would otherwise be Raman inactive. IR spectral signals tend to obtain stronger signals for asymmetric polar bonds, whereas Raman signals are stronger for symmetric, nonpolar bonds (Ali et al. 2013). These techniques have been used to analyze various disease states and can distinguish subcellular structures and biochemical makeup in affected tissues (Krafft et al. 2008). It has been used as a tool for investigating changes in biochemical constituents (e.g., protein content, lipids, carbohydrates, and nucleic acids) (Palaniappan and Pramod 2010) and in differentiating between healthy and diseased tissues (Cheng et al. 2004), and has proven efficacy in toxicological studies (Sivakumar et al. 2014). FTIR has also been used to detect spectral differences between normal and cancerous tissues of the gastrointestinal tract (stomach, colon, and esophagus) (Peng et al. 1998), and has emerged as a useful and accurate tool for the determination of the secondary structure of proteins (Susi and Byler 1986; Surewicz et al. 1993). Attenuated total reflectance (ATR) is a type of FTIR technique that utilizes the properties of evanescent waves to obtain spectra, rather than the transmission method seen in traditional IR spectroscopy, and has been shown in studies to produce high‐quality meaningful spectra to characterize cell and tissue samples (Wong et al. 1995; Andrew Chan and Kazarian 2016).

The objective of this study was to correlate the hemodynamic limitation in the legs of PAD patients, with alterations in the biochemistry of their chronically ischemic leg muscle as determined by ATR‐FTIR micro‐spectroscopy. Our central hypothesis was that broad reaching spectroscopic probing of the ischemic muscle may reveal or identify significant changes to the muscle biochemistry that may be used into identify potential mechanisms of PAD myopathy. The rationale that underlies this research is that spectroscopic techniques provide information‐rich spectral signatures of the biochemistry of the evaluated tissue specimens that is not limited to one or two targets at a time, as is in standard histopathological analyses.

## Materials and Methods

### Tissue samples

The tissue sample collection protocol was approved by the Institutional Review Board of the VA Nebraska‐Western Iowa and University of Nebraska Medical Centers and all subjects gave informed consent. We collected biopsies from the gastrocnemius of four patients with clinically diagnosed critical limb ischemia (CLI) (ABI<0.4), five patients with clinically diagnosed claudication (and 0.4 ≤ ABI<0.9), and four control patients (patients with no lower limb impairment or symptoms of PAD and a resting ABI≥0.9) (Schirmang et al. 2009). Muscle biopsies were collected with a 6 *mm* Bergstrom needle from the anteromedial aspect of the gastrocnemius, approximately 10 *cm* distal to the tibial tuberosity. The biopsies were fixed in methacarn and embedded in paraffin. The muscle samples were cut at 4 *μ*m in cross‐section and mounted on slides for spectral analysis. Demographic data from these patients are given in Table 1.

**Table 1:** Demographics of patients with peripheral artery disease and control patients

	Control	Claudication	CLI^a^
Number of subjects	4	5	4
Mean Age (years) ± Std. deviation	59 ± 7.8	62.8 ± 3.7	59 ± 6.3
Ankle brachial index (ABI)	1.07 ± 0.05	0.6 ± 0.10	0.22 ± 0.09
Sex (male/female)	3/1	5/0	4/0
Obesity	50%	80%	25%
Hypertension	75%	100%	100%
Diabetes mellitus	50%	80%	50%
Smoking	25%	60%	25%

a Critical Limb Ischemia.

### ATR‐FTIR Micro‐spectroscopy data collection

ATR‐FTIR micro‐spectroscopy was used to analyze PAD muscle tissue and establish a framework for a new methodology to identify potential mechanisms of the myopathy. Prior to spectral data collection, the muscle sample slides were deparaffinized with xylene, dehydrated through a series of ethanol washes, and allowed to air dry. Microspectral signatures were collected on a high‐performance FTIR microscope (Smiths IlluminatIR^™^ SensIR) equipped with a microspectroscopy ATR 15× objective accessory with a diamond tip of 100 *μ*m in diameter. ATR‐FTIR microspectral signatures were collected from the fingerprint and functional group regions (600–4000 cm^−1^). A background correction spectrum was collected before each specimen spectrum, 128 scans were collected and averaged to provide a high signal‐to‐noise ratio, and the spectral resolution was set to 4 cm^−1^.

**Figure 1:**
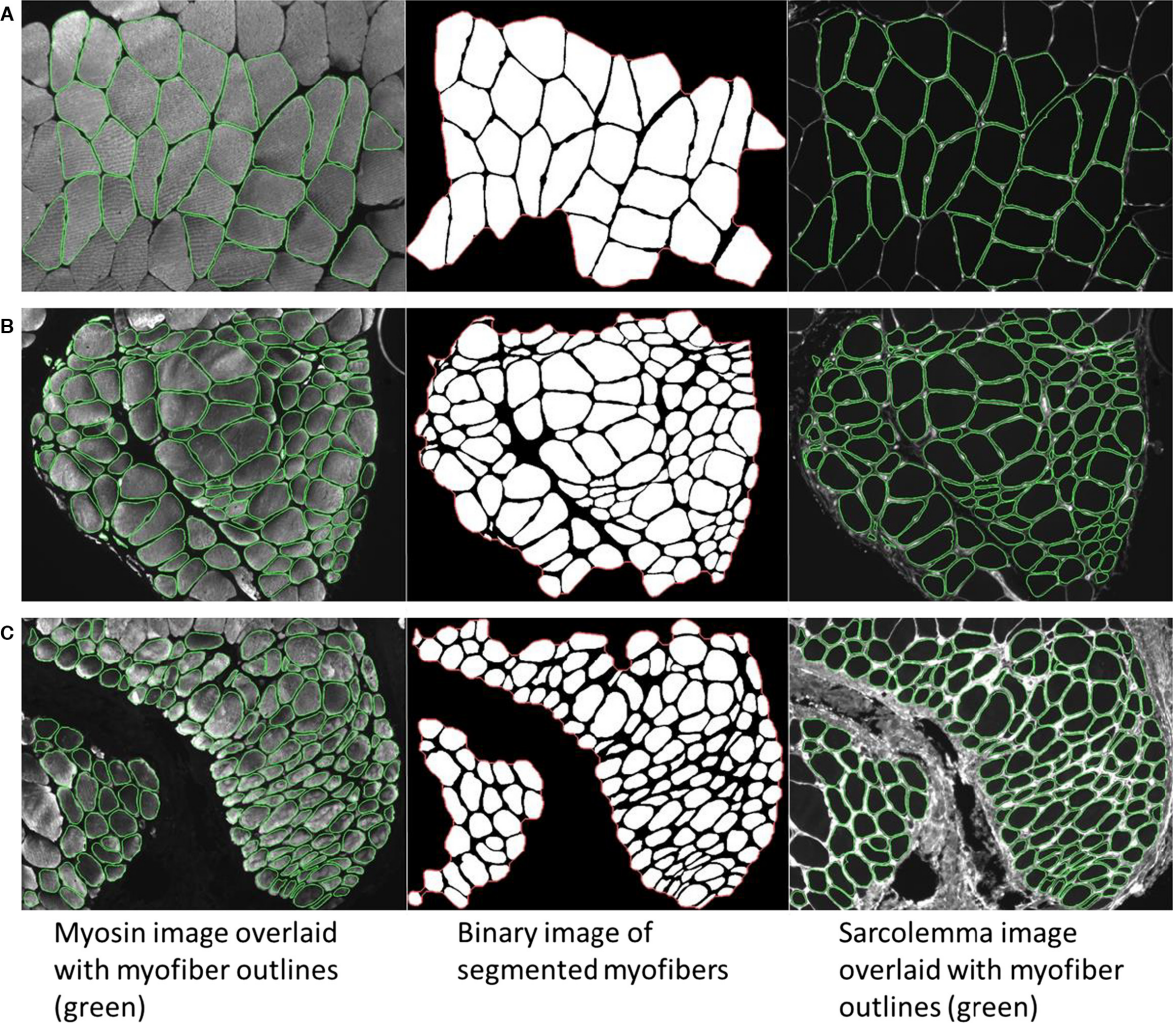
Peripheral artery disease (PAD) muscle displays abnormal muscle morphometry compared to controls (Cluff et al. 2013; Koutakis et al., 2015b). Myofibers were selected for microspectral analysis based on a visual inspection of myofibers with abnormal morphometry. (**A)** Healthy (control) muscle morphometry typically has polygonal shaped myofibers which are uniform in size and shape, and have a thin endomysium and perimysium. (**B)** Muscle tissue from a claudicating PAD patient, and (**C)** muscle tissue from a PAD patient with critical limb ischemia (CLI). PAD muscle displays muscle degeneration with increased variation in size and shape, atrophy and enlargement, round and angular myofibers, and thicker interstitial tissue.

Microspectroscopy allowed spectral data collection from specific locations inside the muscle tissue samples. Microspectral signatures were collected from the center of 10 representative myofibers from each specimen resulting in a total of 130 microspectral signatures. Myofibers were selected based on a visual inspection of myofiber morphology and myopathic characteristics, such as abnormal fiber area, irregular diameter, roundness, fiber density, and clustering of irregular‐shaped myofibers. Figure 1A–C displays abnormal PAD muscle morphology in contrast to healthy muscle shape features (Cluff et al. 2013). In our previous studies, these myofiber morphometrics were objectively defined, correlated with clinical stages of PAD, associated with limb dysfunction, and characterized advancing PAD muscle degeneration (Cluff et al. 2013; Koutakis et al., 2015b). The panels in Figure 1 present myosin labeled images overlaid with myofiber outlines, sarcolemma labeled images overlaid with myofiber outlines, and binary images of the myofiber segmentations.

### Data preprocessing

Data preprocessing included baseline correction, smoothing, and normalization of the raw ATR‐FTIR microspectral signatures. Baseline correction and smoothing were performed to remove additive noise and are essential steps in spectral biosignal processing (Chen et al. 2004; Sweeney et al. 2012; Cluff et al. 2016). The spectral data were smoothed using a 15‐point box‐car smoothing algorithm. A baseline correction algorithm was developed in Matlab software (R2015b, The Mathworks Inc., MA) to iteratively optimize a piecewise cubic interpolation of the raw ATR‐FTIR spectra.

Normalization was done in order to remove multiplicative error and put the data on the same scale to allow comparisons of the results and relationships among spectra. Although there are several approaches to normalizing spectral data, two common methods are multiplicative scatter correction (MSC) and standard normal variate (SNV) normalization (Rinnan 2009; Azzalini et al. 2012). We normalized the ATR‐FTIR spectra using the standard normal variate (SNV) technique because it is designed to remove multiplicative error and preserve the linear relationship between the spectral signal and sample concentration (Barnes et al. 1989; Rinnan 2009).

### Data analysis

Standard univariate statistical analysis of variance (ANOVA) followed by a Bonferroni adjusted multiple comparison analysis was performed on four prominent regions in the spectra to assess if there were statistically significant differences in the spectral profiles. The significance level was set as *α* = 0.05. Additionally, a multivariate partial least squares regression (PLSR) model was developed using the entire spectral region (600 *cm* ^*−1*^ to 4000 *cm* ^*−1*^) to predict patient ABI's. The PLSR algorithm combines techniques used in principal component analysis and multiple linear regression and attempts to quantify the strength of the relationship between the response variable and a set of predictor variables (Geladi and Kowalski 1986). PLSR searches for principal components (called factors) that are orthogonal to each other (i.e., independent) and tries to relate them to the response variable. The variation present in the predictor variables (spectra) can be summarized into a few PLS factors. Decomposition of the PLS factors, using the loading vectors, can identify which variables had the most weight in the predictive PLSR model. A 10‐fold cross‐validation, which is a common statistical analysis technique for estimating model performance, was performed on the data set to evaluate the performance of the predictive model. Cross‐validation is a standard multivariate statistical technique often used on small data sets to assess model stability and determine how well it will perform on future data sets (Hastie et al. 2009). The cross‐validation technique rotates the membership of the samples (during calibration) to ensure that the results are not membership dependent (i.e., calibration group and validation group) and to ensure that the model is not overfitting the data.

## Results

**Figure 2:**
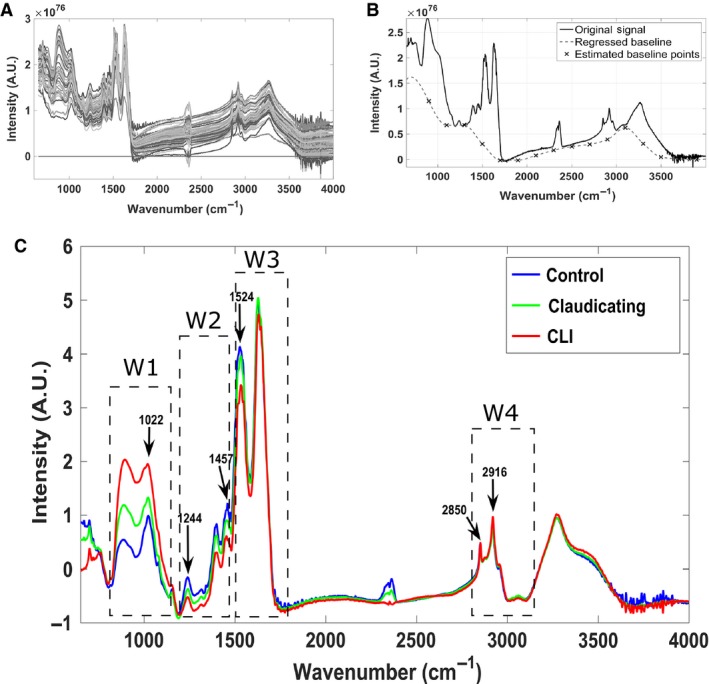
(**A)** All raw ATR‐FTIR Spectra collected from Peripheral artery disease (PAD) patients and controls. (**B)** Spectral data preprocessing included baseline correction using a fourth degree fitted polynomial and normalization. (**C)** Average control, claudicating, and critical limb ischemia (CLI) spectral signatures; Windows, W1: phosphate & oligosaccharide region (900–1200 cm^−1^), W2: mixed region (1200–1500 cm^−1^), W3: protein/peptide region (1500–1700 cm^−1^), W4: fatty acid region (2800–3000 cm^−1^).

Figure 2A presents the raw ATR‐FTIR spectra obtained from the muscle samples (i.e., 10 spectra per sample resulting in a total of 130 spectra). The baseline correction algorithm estimated baseline regression points (Fig. 2B) and subtracted the fitted multipolynomial curve to remove intrinsic autofluorescence background signals and improve signal‐to‐noise ratios (Lieber and Mahadevan‐Jansen 2003; Afseth et al. 2006; Zhao et al. 2007; Beier and Berger 2009). Figure 2C presents four major absorption regions that were investigated: Window 1 (W1) 900–1200 cm^−1^ (oligosaccharides and phosphate group region), Window 2 (W2) 1200–1500 cm^−1^ (mixed region of fatty acids, proteins, nucleic acids, and phosphate groups), Window 3 (W3) 1500–1700 cm^−1^ (protein/peptide region), and Window 4 (W4) 2800–3000 cm^−1^ (fatty acid region) (Payne and Veis 1988; Naumann et al. 1991; Wang et al. 1993; Packer 1994; Rehman and Bonfield 1997; Baranska 2013).

**Figure 3:**
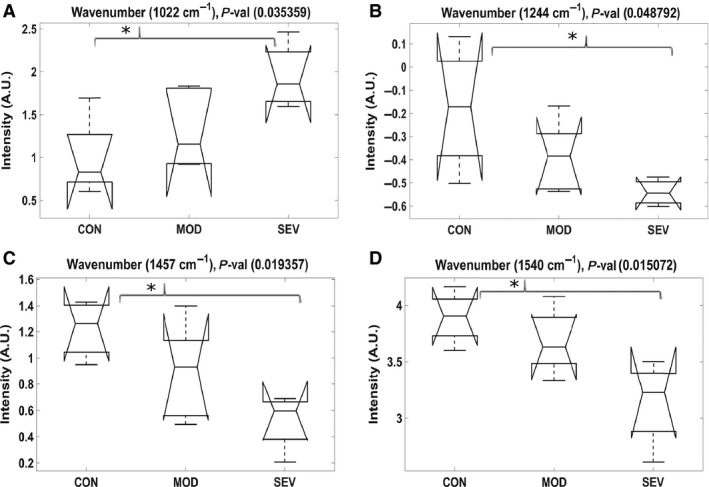
(A–D) Notched box‐and‐whisker plots representing four significant peaks (*P* < 0.05) identified by the ANOVA between control (CON), claudicating (CLAUD), and critical limb ischemia (CLI) Peripheral artery disease (PAD) patients, followed by a Bonferroni adjusted multiple comparisons analysis indicating which groups were significantly different. In the box‐and‐whisker plot, the center line indicates the median, while the top and bottom of each box represents the 75th and 25th percentiles, respectively. The notches represent the 95% confidence interval around each median, and the upper and lower whiskers indicate the maximum and minimum values, respectively.

After performing an ANOVA and a Bonferroni adjusted multiple comparisons analysis on these regions, there were significant differences (*P < 0.05*) in wavenumbers 1022 (*P = 0.035*), 1244 (*P = 0.048*), 1246 (*P = 0.048*), 1457 (*P = 0.019*), 1526 (*P = 0.036*), 1540 cm^−1^ (*P = 0.015*) across controls, claudicating, and CLI PAD muscle. Figure 3A–D presents notched box‐and‐whisker plots of four of these wavenumbers. These differences signify important changes in the biochemical composition of the muscle cells as they sustain damage and eventually undergo apoptosis or necrosis, with concomitant change to the muscle pathology. While they are not measured with ABI, many of these changes may be strong candidates as spectral biomarkers in patients with PAD.

**Figure 4:**
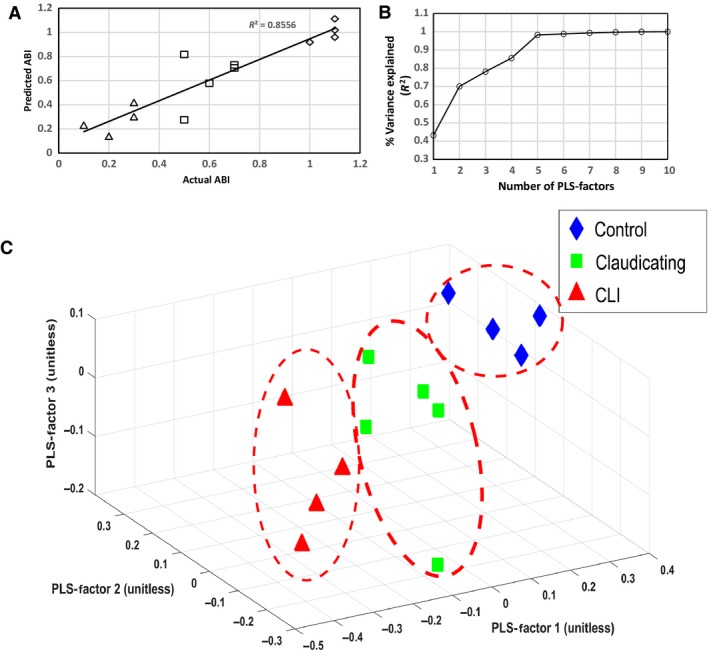
(**A)** The partial least squares regression (PLSR) model was able to predict patient ABIs with an *R* ^2^=0.85 using the first four PLS factors and a 10‐fold cross validation (**B)** The first 3‐4 PLS factors explained most of the variance in the data set. (**C)** A 3D plot of the first 3 PLS factors demonstrates clear separation between controls, claudicating, and CLI patients based on the spectral profiles of the muscle biochemistry.

**Figure 5:**
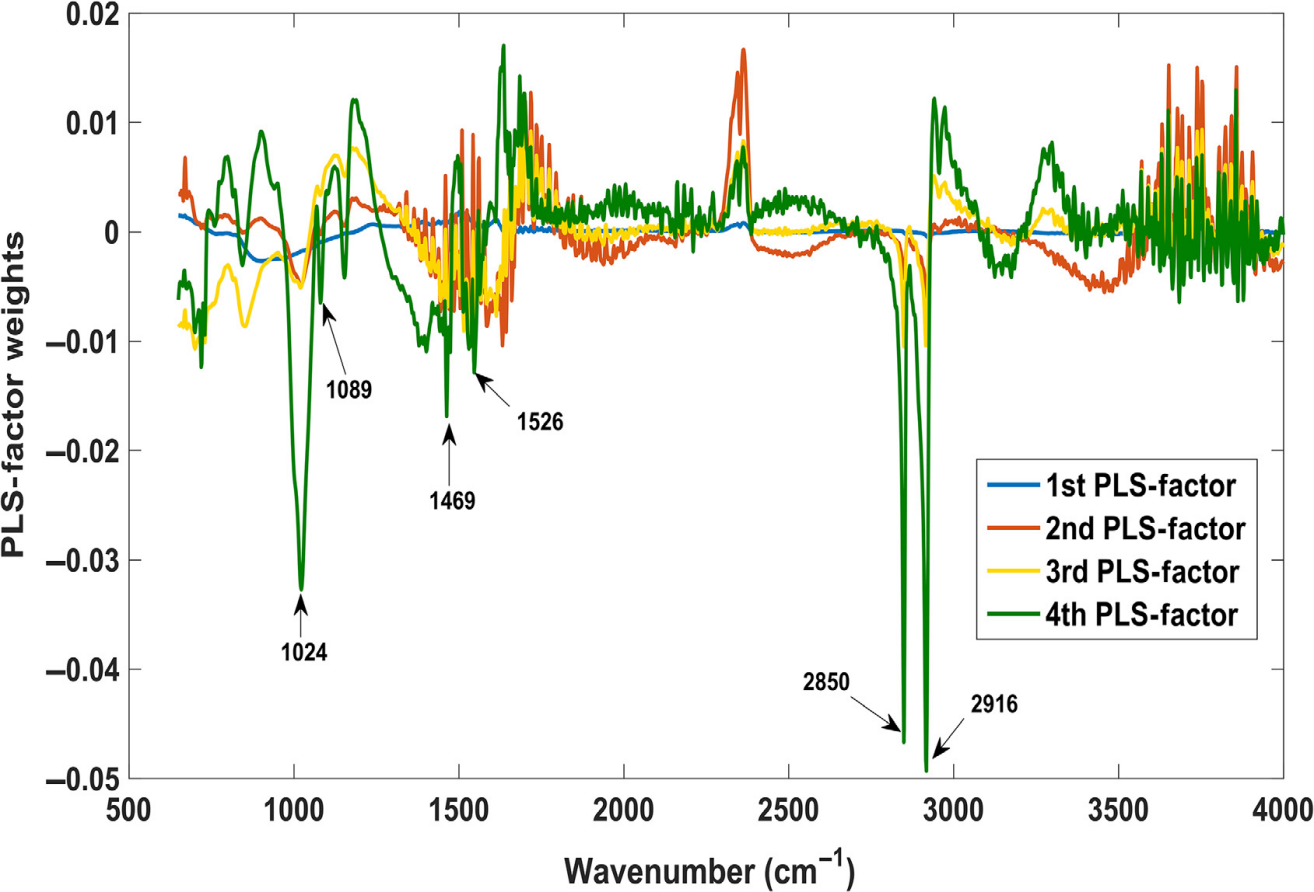
The weight of each PLS‐factor indicates which wavenumbers had the most influence on the partial least squares regression (PLSR) predictive model. This was used to identify important bands and focus the interpretation of the spectral signatures to potential biochemical change.

Using the first four PLS factors, the PLSR model was trained and the predictive performance was evaluated using a 10‐fold cross‐validation procedure. The PLSR model was able to predict patient ABIs with a coefficient of determination of *R* ^2^ = 0.85 during cross‐validation (Fig. 4A) and a root mean square error‐cross validation (RMSE‐CV) of 0.37. Figure 4B demonstrates that most of the variance in the data was explained by the first three to four PLS factors. A 3D plot (Fig. 4C) of the first three PLS factor scores demonstrate clear separation of the controls, claudicants, and CLI patients. An examination of the PLS factor weights (Fig. 5) gives an indication of which wavenumbers had the most important impact on predicting the patient's ABIs, thus indicating that these are key spectral bands.

These key spectral bands may point the way toward new or neglected therapeutic targets. Accordingly, wavenumbers 1024, 1089, 1469, 1526, 2850, and 2916 cm^−1^ were the strongest spectral biomarkers for ABI prediction. It was interesting to note that some of these key spectral bands in the PLSR multivariate analysis, also matched some of the bands found to be significant in the univariate (ANOVA) statistical analysis.

## Discussion

In this study, we demonstrated that ATR‐FTIR microspectroscopy can perform detailed and precise measurements of the biochemical changes that occur in the chronically ischemic muscles of PAD patients. This is the first study to produce ATR‐FTIR profiles of normal and diseased skeletal muscle and our data demonstrate that the skeletal muscle is very sensitive to chronic ischemia and undergoes biochemical changes that correspond to the degree of hemodynamic compromise and the clinical severity of PAD.

The ATR‐FTIR analysis revealed spectral bands in the phosphate region (Window 1: 900–1200 cm^−1^) showing a higher intensity in both claudicating and CLI patients, signifying an increase in intracellular phosphate with increasing muscle damage. This may be a critical observation, considering the importance of phosphate‐related molecules in cellular health and metabolism. The bands at 1089 and 1469 cm^−1^ are attributed to phosphate‐oxygen (P‐O) stretching present in sphingosine‐1 phosphate, a biosignaling phospholipid that serves an important role in muscle regeneration (Garcia‐Pacios et al. 2009). Research has shown that this lipid can activate satellite cells which can differentiate to increase muscle regeneration (Donati et al. 2013). The increase in the sphingosine‐related band suggests that the ischemic myofibers increase the phosphorylated (activated) sphingosine possibly in an effort to maintain/regenerate the myofibers in response to damage produced by ischemia in the PAD limbs (Saiardi 2012; Donati et al. 2013). The increased phosphate levels could also be due to bone resorption, which releases both phosphate and calcium into surrounding tissues, and could indicate damaged hormone regulation pathways. Bone resorption has been shown to be linked to cardiovascular diseases including PAD, though there has been little research done on how it affects muscle damage (Farhat et al. 2007; Farhat and Cauley 2008; Collins et al. 2009). One of the more interesting observations is that some key signatures (1022, 1024 cm^−1^) in the muscle spectral fingerprints indicate an increase in glycogen levels (Wood et al. 1996, 1998; Movasaghi et al. 2008). It is possible that in the chronically ischemic state, PAD muscles attempt to compensate for the blood flow restriction by increasing the resting glycogen stores in the muscle. This finding is similar to those of Askew et al. (2005) showing that muscle glycogen content did not differ between PAD subjects and controls. It is possible that the increased glycogen levels are related to an increase in type II myofibers in the specimens we sampled as Askew et al. (2005) have shown that glycogen levels are higher in fast versus slow type myofibers.

The decrease in intensity observed in the mixed region (Window 2: 1200–1500 cm^−1^) of the PAD spectra compared to the controls may be attributed to a decrease in deoxyribonucleic acid (DNA) content in the cell. The bands contributing to wavenumbers 1244 and 1246 cm^−1^ have been attributed to asymmetric phosphate (PO_2_) stretching present in DNA (Fabian et al. 1995; Wood et al. 1996, 1998; Fukuyama et al. 1999; Dovbeshko et al. 2002). Additionally, decreases in the peptide region (Window 3: 1500‐1700 cm^−1^), namely, the band surrounding 1524 cm^−1^ has been found to be related to carbon‐nitrogen (C=N) and carbon‐carbon (C=C) stretching present in guanine (Fabian et al. 1995; Wood et al. 1998; Dovbeshko et al. 2000). It is possible that these findings reflect an overall decrease in DNA level in the cells and that this is related to damage of nuclear and mitochondrial DNA from the chronic ischemic process (Bhat et al. 1999; Pipinos et al. 2006). DNA fragmentation that occurs during apoptosis or as a result of the increased oxidative damage seen in PAD muscle may be contributing to this finding (Pipinos et al. 2008; Weiss et al. 2013; Koutakis et al. 2014). Furthermore, significant damage to the mitochondria and their DNA has been previously shown in PAD (Ryan et al. 2015), and may be the reason behind the damage to DNA, which appears to be present in the ATR‐FTIR spectra (Makris et al. 2007; Pipinos et al. 2007; Koutakis et al., 2015a; Thompson et al. 2015).

The band occurring at 1457 cm^−1^ is attributed to bending of protein methyl groups, and the band occurring at 1540 cm^−1^ is considered to be an amide II band (Gazi et al. 2003; Fujioka et al. 2004), both of which indicate a decrease in intracellular protein content in the PAD spectra compared to the controls. Research from our group has shown significant damage to the proteins of the myofibers including proteins that form the cytoskeleton of the fiber (Koutakis et al., 2015a; Thompson et al. 2015). Our research has also shown that decreased protein concentration (normalized to muscle wet weight) in the calves of PAD patients is associated with worsening myopathy and most importantly predicts a significant decrease in 5‐year mortality rate of the patient (Thompson et al. 2015). Our current findings are also in agreement with work from other groups showing that muscle injury results in leakage of myofiber proteins with increases in the levels of several different muscle proteins in the serum, and their detection is one of the primary laboratory methods used for the identification and diagnosis of muscle damage in hospitalized patients (Brancaccio et al. 2010). Creatine kinase (CK) is one of the proteins easily detected in association with muscle injury (Baird et al. 2012) while other muscle proteins constituting some of the most essential structures of the muscle cell, including actin and myosin are also detected in the serum of patients having different types of muscle damage (McKune et al. 2014).

Further investigation of the spectra reveals a marked decrease in intensities of overall lipids in the fatty acid region (Window 4: 2800–3000 cm^−1^) in PAD muscle compared to controls. The bands occurring at 2850 cm^−1^ and 2916 cm^−1^ have been attributed to cholesterol and creatine, which had lower intensity peaks in PAD muscle (Dovbeshko et al. 2000). It is known that creatine kinase levels are increased in the blood, a result of leakage from damaged muscle cells (Totsuka et al. 2002; Friedman et al. 2003; Baird et al. 2012). The function of CK is to regulate the ratio of creatine: phosphocreatine to provide an acute supply of phosphate for use in ATP synthesis for cell energetics. CK, creatine, phosphocreatine, and AMPK have been shown to be involved in complex interactive regulatory processes (Ponticos et al. 1998). Thus, a reduction in creatine levels could have profound effects on AMPK and CK activity and the mechanisms they regulate, such as glucose uptake and ATP synthesis. The differences in this region could also be an indicator for lipid peroxidation changes of membrane lipids. The peroxidation of phospholipids could play an integral role in the total lipid decrease in muscle tissue. Many phospholipids, when cleaved, produce fatty acids such as arachidonic acid. Arachidonic acid causes inflammation, which is increased in damaged muscle tissue. Sphingosine is also a component of many phospholipids; cleavage of these phospholipids would aid in the synthesis of sphingosine‐1 phosphate, which would ultimately help regenerate lost muscle tissue. The decrease of intramyocellular lipid (INML) content could also be a result of muscle damage. Little research has been done on INML in damaged PAD muscle, but the well‐described lipid peroxidation occurring within the myofibers can not only cause oxidation of these lipids but also initiate pathways that will lead to export of the oxidized lipid domains and lipid fragments from the cell (Schrauwen‐Hinderling et al. 2003, 2006; Powers and Jackson 2008). Furthermore, little research has been done on the concentrations and activity of peroxidative enzymes such as those of the peroxisomes during chronic muscle injury. These could be the key components in the regulation of lipid bilayer homeostasis in ischemic conditions and warrant further research into the role they play in PAD.

While ATR‐FTIR provides broad reaching and information‐rich spectral profiles of the muscle biochemistry limitations of this study include the small patient sample size and that the interpretation of the spectral peaks can be complicated. Additionally, although the myofibers were selected based on visual inspection to obtain spectra from, there is the possibility that the results may not be representative of the entire muscle sample due to the small sample size of myofibers measured. Interpreting the spectral profile is complicated because the optical spectral signals are frequently the aggregate of signals from many biomolecules present in the muscle samples. Further, the spectral peaks that were identified and discussed in this study warrant further study to provide supporting biochemical analysis to validate these potential targets. As the experience of our team and others increases with the specificity and analysis of each one of the windows and bands of ATR‐FTIR, we will be able to provide detailed dissections of the biochemical effects of PAD on human myofibers. While many of these effects and mechanisms have been highlighted, more research is needed to further elucidate the fundamental causes of ischemia‐induced muscle degeneration, and to accurately grade it both for the purposes of measuring the degree of myopathy in the PAD leg and for using it as a method to follow the responses of the muscle to different treatment methods. Further work from our group endeavors to produce chemical maps for entire cross sections of PAD muscle. By mapping spectral signals from the entire cross section of a myofiber, more insight could be obtained from the peaks that were identified in this study. ATR‐FTIR may be a powerful bioanalytical technique to provide critical insight into potential spectral biomarkers for muscle damage and how it affects the tissues at a molecular level. Muscle cell physiology is an expression of numerous complex mechanisms, many still unknown, that could be used to develop new therapies or tests for early diagnosis and more effective treatment for PAD‐induced muscle damage.

## Significance and Conclusions

ATR‐FTIR spectroscopy was able to characterize the effects of PAD on the gastrocnemius muscle by identifying the unique biochemical signatures of the affected myofibers. The identified signatures can discriminate control spectra from PAD muscle tissue and correlate with the clinical presentation of PAD patients. While the spectra do not immediately reveal mechanisms operating to connect the arterial occlusive disease with the myopathy of PAD, they point into areas of research that need to be explored, and play an integral role in developing methods for discovering new therapeutic targets for treating the muscle damage in the legs of PAD patients. This work may also identify novel spectral biomarkers that can complement the currently available methods for the diagnosis of the degree of myopathy and for monitoring the way the myopathy responds to established and new therapies for PAD patients.

## Conflict of Interest

None declared.
